# A Giant Left Coronary Button Aneurysm After Aortic Root Remodeling Procedure in a Patient With Marfan Syndrome: A Case Report

**DOI:** 10.7759/cureus.26031

**Published:** 2022-06-17

**Authors:** Akihiro Higashino, Tsuyoshi Taketani, Hiroyuki Suzuki, Sumio Miura, Takayuki Ohno

**Affiliations:** 1 Cardiovascular Surgery, Mitsui Memorial Hospital, Tokyo, JPN; 2 Cardiovascular Surgery, Okayama University, Okayama, JPN

**Keywords:** angiotensin-ii type-1-receptor blocker, aortic root replacement., piehler's technique, coronary button aneurysm, marfan syndrome

## Abstract

Coronary button aneurysm is a well-known complication of aortic root surgery, especially in patients with Marfan syndrome.

We present a case of a giant left coronary button aneurysm that occurred 20 years after an aortic root remodeling procedure was performed. A 32-year-old man with Marfan syndrome underwent the aortic root remodeling procedure for annuloaortic ectasia. Thirteen years later, an aortic aneurysm with chronic aortic dissection was diagnosed, and partial aortic arch replacement was performed. Twenty years after the first procedure, a 73-mm left coronary button aneurysm was observed. Due to dense adhesions from repeated surgeries, we approached the aneurysm through the artificial graft lumen, and the coronary artery was successfully reconstructed using Piehler's technique.

When performing aortic root surgery for Marfan syndrome, the risk of coronary artery button aneurysm formation should be considered. Once an aneurysm is formed, a surgical strategy that assumes dense adhesions is essential.

## Introduction

Aneurysmal change of the residual aortic wall is a severe complication of aortic root surgery. Since the development of surgical techniques, such as various coronary artery reconstruction methods (Carrel patch, Cabrol, and Piehler’s methods), complications at the coronary artery reconstruction site have decreased [1]. However, coronary button aneurysm formation has been reported in patients undergoing coronary artery reconstruction using the Carrel patch technique, especially in patients with Marfan syndrome (MFS). Some residual aortic walls are considered to form an aneurysm owing to their connective tissue abnormalities, such as medial cystic necrosis [2,3]. Herein, we report a case of a rapidly growing giant coronary button aneurysm in a patient with MFS. We successfully treated it despite the presence of solid adhesions due to repeated surgeries.

## Case presentation

**Figure 1:**
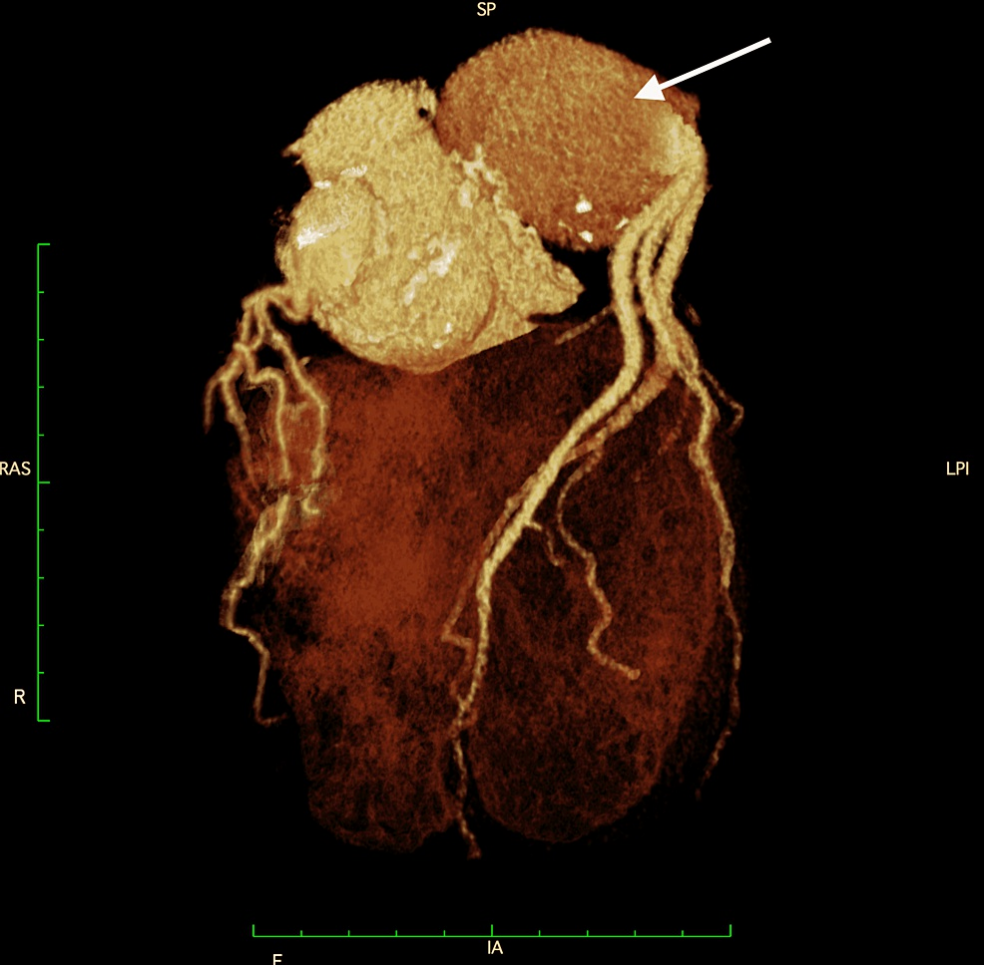
Chest computed tomography before the third surgery. Chest computed tomography before the third surgery. The left coronary button aneurysm enlarged up to 73 mm in diameter, while the right coronary button aneurysm was slightly enlarged.

A 32-year-old man with MFS underwent valve-sparing aortic root replacement (Yacoub procedure) for annuloaortic ectasia in the US. At the age of 45 years, DeBakey type-II chronic aortic dissection was identified, and he underwent partial arch replacement at our institute. The patient was prescribed losartan to prevent aneurysm formation. However, he had stopped going to the outpatient clinic when he was 49 years old and had not taken losartan since then. Bilateral coronary button aneurysms had already been pointed out at that time. The left coronary button aneurysm was oval-shaped with a diameter of 25 mm, while the right aneurysm was 15 mm, with no change in size from the CT four years before, and both showed no tendency to increase. Three years later, the patient presented to the emergency room with complaints of chest pain. Computed tomography revealed that the left coronary button aneurysm had grown to 73 mm in diameter (Figure 1) and that there was no stenosis in the coronary artery. Since there were no signs of myocardial ischemia on blood tests or electrocardiogram, the cause of the chest pain was considered to be the enlarged aneurysm. In addition, echocardiography revealed moderate aortic regurgitation due to aortic annulus dilatation. We believed it would be difficult to repair the aortic regurgitation and that it would worsen in the future if untreated. We scheduled a left coronary artery reconstruction and aortic valve replacement. 

**Figure 2:**
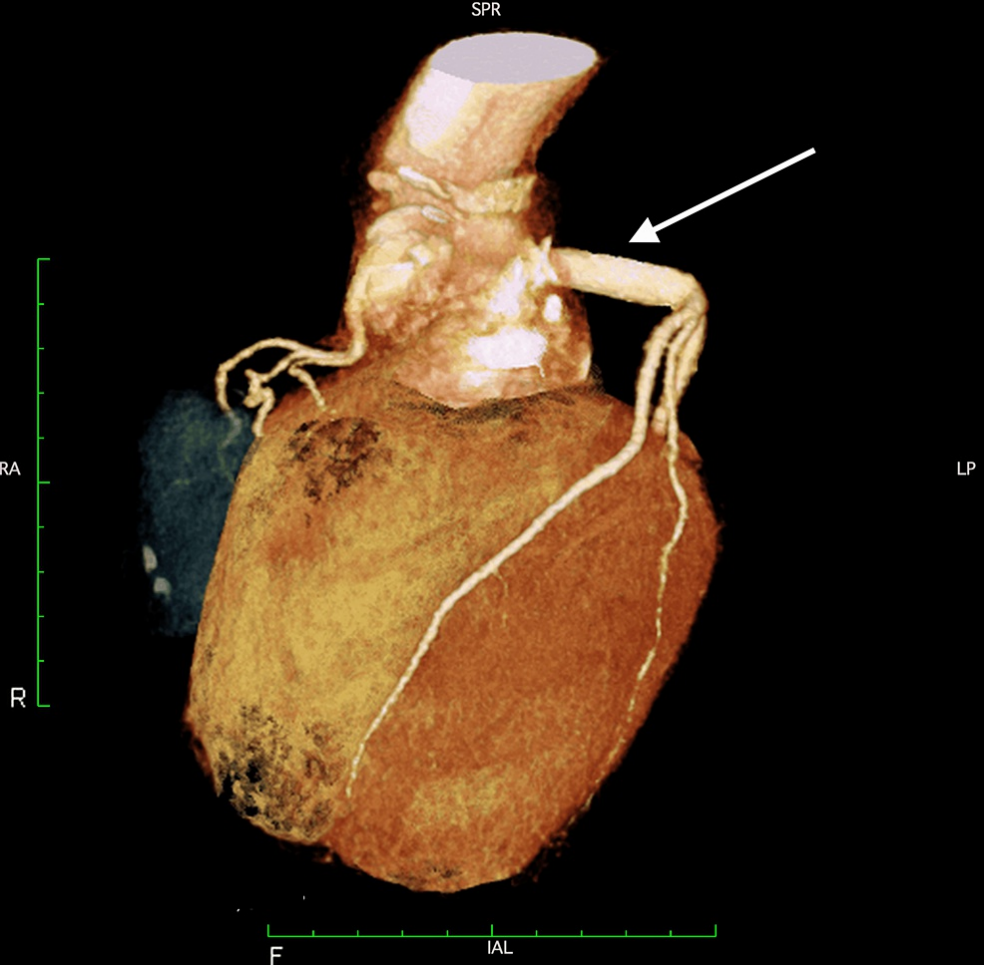
A postoperative contrast-enhanced computed tomography The coronary artery was reconstructed with the artificial graft (arrow) without any complications of the anastomosis site.

After the third median sternotomy was performed, cardiopulmonary bypass (CPB) was established with an arterial cannula in the right femoral artery and a venous drainage cannula in the right femoral vein. Under mild hypothermia (28°C), the ascending aortic graft was clamped, and cold crystalloid cardioplegia was administered. It was challenging to identify the coronary aneurysms from the outside because of tight adhesions around the graft. Therefore, we decided to approach the aneurysm from the ascending aortic graft. The anterior surface of the graft was incised to identify the anastomosis of the coronary button. The anastomosis site of the coronary button, i.e., the neck of the aneurysm, was about 25 mm in diameter. It was incised transversely and enlarged, and a thrombus in the aneurysm was removed to identify the ostium of the left main trunk (LMT). Four mattress sutures were placed on the rim of the LMT using 4-0 Prolene with a felt pledget, and distal anastomosis was performed with an 8-mm Dacron graft. The proximal side of the graft was anastomosed to the buttonhole of the aortic graft with 4-0 Prolene running suture, stretching the graft sufficiently forward to avoid graft kinking. Subsequently, aortic valve replacement was performed using a mechanical valve. The right coronary button was not tending to enlarge but was forming an aneurysm, so to reduce the possibility of future enlargement, it was sutured using a 4-0 Prolene mattress suture with felt pledgets. Weaning from the CPB was successful without any complications. Postoperative recovery was uneventful. The postoperative contrast-enhanced computer tomography (CT) showed no kinks in the graft and no other significant problems (Figure 2). The patient was discharged on postoperative day 19.

## Discussion

Compared to the original Bentall procedure, surgical techniques have improved, and complications such as coronary button aneurysms have decreased [1]. However, coronary button aneurysms are frequently reported in patients with MFS [4]. Kazui et al. suggested that the remaining aortic wall may be involved in the formation of coronary button aneurysms in many cases of MFS. The incidence of coronary artery button aneurysms is lower with a smaller-diameter coronary artery button [3]. In this case, a felt strip was used to reinforce the anastomosis of the coronary button. Nevertheless, the size of the coronary button was relatively large (25 mm in diameter), which could have caused the aneurysm. 

Therefore, the coronary button should be as small as possible to reduce the remaining aortic wall. In addition, to prevent pressure from being applied to the remaining aortic wall, the suture should be applied to the rim of the coronary artery ostium rather than the remaining aortic wall when anastomosing the coronary button.

Several surgical procedures for coronary aneurysms have been reported, such as direct suture of the coronary artery and aortic wall, coronary artery replacement, and coronary artery bypass surgery; however, none of these procedures have been standardized [5,6]. Since surgery for coronary artery button aneurysms is always a redo open heart surgery, it is vital to perform a surgical strategy that considers adhesions. In this case, since it was the third open-heart surgery and dislodging the adhesions around the coronary aneurysm was expected to be difficult, we chose to approach the aneurysm from the graft lumen by cutting through the graft. Moreover, the coronary artery was reconstructed using a modified interposing method [5]. There are three advantages to this approach.

1. It does not require excessive detachment of adhesions and avoids intraoperative injuries.

2. It allows reconstruction with the original blood flow direction.

3. It can be applied to large coronary aneurysms because mobilization of the coronary ostium is not required.

We believe that this is a very useful method; however, the length of the graft needs to be carefully selected to avoid bending.　

It may be also important to prevent aneurysm formation by using losartan. Aneurysm in a patient with MFS is reported to be associated with increased transforming growth factor-β signaling and can be prevented by an angiotensin-II type-1-receptor blocker losartan [7,8]. Effect of ARBs on aortic root growth rate in patients with MFS showed variable results, but losartan could improve event-free survival in patients with MFS after >8 years of follow-up [9]. In the present case, the coronary button aneurysm grew rapidly from 25 to 73 mm in three years.

This enlargement was observed after the losartan withdrawal. Considering that the abnormal aortic wall remains in patients with MFS, including the coronary button, it is advisable to continue losartan and regular CT follow-up even after aortic root replacement. 

## Conclusions

We can conclude that the intra-aortic approach was useful for treating coronary button aneurysms with advanced adhesions. When reconstructing coronary arteries, especially in patients with MFS, it is important to allow the least aortic wall length and prevent aneurysm formation by continuing angiotensin-receptor blocker medications.
